# The Effect of Religion on Candidate Preference in the 2008 and 2012 Republican Presidential Primaries

**DOI:** 10.1371/journal.pone.0152037

**Published:** 2016-04-04

**Authors:** Leigh A. Bradberry

**Affiliations:** Department of Political Science, California State University, Northridge, Los Angeles, California, United States of America; University of Amsterdam, NETHERLANDS

## Abstract

Thanks to the work of politics and religion scholars, we now know a lot about the relationship between religion and voting in American presidential *general* elections. However, we know less about the influence of religion on individual vote choice in presidential primaries. This article fills that gap by exploring the relationship between religion and candidate preference in the 2008 and 2012 Republican primaries. Using pre-Super Tuesday surveys conducted by the Pew Research Center, I find that the Republican candidate who most explicitly appealed to religious voters (Mike Huckabee in 2008 and Rick Santorum in 2012) was the preferred candidate of Republican respondents who attended religious services at the highest levels, and that as attendance increased, so did the likelihood of preferring that candidate. I also find that identification as a born again Christian mattered to candidate preference. Specifically, born again Christians were more likely than non-born again Christians to prefer Huckabee to Mitt Romney, John McCain and Ron Paul in 2008, and Santorum to Romney in 2012. Although ideology was not the primary subject of this article, I find that ideology was also a statistically significant predictor of Republican candidate preference in both 2008 and 2012. This robust finding reinforces scholars’ prior work on the importance of ideology in explaining presidential primary vote choice. The overall findings of the paper provide evidence that religion variables can add to our understanding of why voters prefer one candidate over another in presidential primaries.

## Introduction

Thanks to the work of religion and politics scholars over the last three decades, the connection between religion and vote choice in American presidential *general* elections is well documented (e.g., [1], [2], [3]). One consistently replicated finding is that while religious affiliation or “belonging” alone used to be the main dividing line when Americans went to the polls, the degree of an individual’s religious *commitment*—typically measured by looking at the individual’s behavior and beliefs—is now a key predictor of vote choice in general presidential elections. One of the most reliable measures of that commitment is attendance at religious services, and over the last three decades, increased attendance (among whites) is highly consistent with more support for the Republican candidate in the general election [2]. And while religious affiliation in and of itself is no longer *the* most important factor, it remains the case in recent presidential elections that whites who identify as born again or evangelical Protestants support the Republican candidate by increasingly wide margins [2].

However, despite the fact that we now know a lot about religion and voting in presidential general elections, little is known about the effect of religion on vote choice in presidential *primaries*; how, for example, might a voter’s religiosity or religious identification affect her decision when candidates within parties are not divided as sharply by ideology and issue positions? In particular, given that high-attending voters and white born again or evangelical Protestant voters overwhelmingly prefer the Republican candidate in the presidential general election, how might those same voters sort themselves in a Republican presidential primary, when the party label is not there to help them?

This paper provides some preliminary answers to that question, using the unique opportunity provided by the 2008 and 2012 Republican primaries. Neither year featured an incumbent Republican president, so the field was wide open. In addition, the final contenders each year brought an interesting mix of religious diversity to the table. Just prior to Super Tuesday—the important election day early in the primary cycle on which the largest number of states hold their primary elections—the last-remaining choices in 2008 included: Mike Huckabee, a Baptist minister who is proudly an evangelical Christian; John McCain, a well-known veteran politician who is Protestant but not perceived as particularly religious [4]; Mitt Romney, the first serious contender for president who is a member of the Church of Jesus Christ of Latter-day Saints (also known as the Mormon faith); and Ron Paul, who is Protestant, but whose message was far more focused on economic and foreign policy issues than on religion or “moral values” issues.

Although Romney came in third in 2008, he emerged as the front-runner in 2012. The other last-remaining candidates in the Republican field prior to Super Tuesday included: (1) Rick Santorum, a Catholic who is known for being very socially conservative and vocal about his religious beliefs, and is thus in many ways more like a born again or evangelical candidate; (2) Newt Gingrich, the former House Speaker who is a converted Catholic; and (3) Ron Paul, who once again rounded out the final four. In sum, given the absence of an incumbent president, the religious diversity among the candidates, and the presence of the first viable Mormon candidate in both years, the 2008 and 2012 Republican primaries present the perfect opportunity to investigate how religion might affect candidate preference in Republican presidential primaries.

In this article, I add to the extant literature on religion and politics in the American context by presenting evidence that religion is not simply relevant to vote choice in presidential general elections, but can also help explain Republican presidential *primary* vote choice. These findings also contribute to prior literature that seeks to explain individual vote choice in presidential primaries. Using surveys from the Pew Research Center taken just before Super Tuesday in 2008 and in 2012, I find that highly religious Republican voters (as measured by attendance at religious services) preferred the candidate in each election who most explicitly appealed to religious voters, and that as attendance increased, so did the likelihood of preferring that candidate (Huckabee in 2008 and Santorum in 2012). Similarly, I find that Christian respondents who self-identified as born again or evangelical were more likely than those who were *not* born again or evangelical to prefer Huckabee to the other candidates in 2008 and Santorum to Romney in 2012.

In light of the existing literature discussed below, it is important to state up front what this article is, and what it is not. This article is not an attempt to provide a comprehensive explanation of the entire nomination process, which now takes place over almost an entire year—from the first candidates’ announcements in the summer of the year preceding any actual voting to the last primaries in June of the presidential election year. As the title of Bartels’ [5] important work on primaries indicates, the primaries process is dynamic, and a single pre-Super Tuesday survey obviously cannot capture voters’ changing perceptions of a candidate’s chances of winning the nomination as the primary process unfolds (see also [6] and [7]), nor can it capture the potential influence of party elites in narrowing down the candidates that voters have to choose from on Super Tuesday [8]. Rather, the goal of this article is modest, but important: to provide concrete evidence that religion can add to our understanding of why individual voters prefer one candidate over another when choosing among the candidates presented to them in presidential primaries, and in this particular case, in Republican primaries. This article therefore contributes to the literature on the variables that are worth considering when explaining presidential primary vote choice at the individual level.

## Prior Work on Vote Choice in Presidential Primaries

The context of presidential primaries is very different from that of general elections. First, a voter facing a decision in a presidential primary election lacks the one heuristic most useful to voters in a general election: a party label. In the general election, American voters have two main choices: the candidate representing the Republican party and the candidate representing the Democratic party. However, an individual who intends to vote in either the Democratic or Republican primary is faced with multiple candidates from the *same* party. To use the most recent 2016 primaries as an example, there initially were five candidates for the Democratic nomination and seventeen candidates for the Republican nomination. Second, candidates *within* the same party typically do not differ as much on issues as do candidates from different parties. For example, Republican candidates are more likely to have views on abortion that are very similar to each other, but very different from the Democratic candidates. And finally, unlike the general election which takes place on a single day, primaries take place over an extended period of time; in 2008 and 2012, the first primary was in early January, and the last primary was in June. As a result of these major differences between presidential primaries and the presidential general election, theories about what motivates individual vote choice in general elections are not easily transferrable to the primary context.

Given these differences, and particularly the absence of the powerful explanatory variable of party identification (the strongest predictor of voting behavior in the presidential general election), a comprehensive explanation of individual vote choice in presidential primary elections has proven elusive. Some of the initial variables considered and tested include candidate qualities [9], issues/issue positions [5], [9], [10], [11], electability and/or viability [6], [12], [13], and ideology [9], [11]. However, none has emerged as *the* dominant variable in terms of explanatory power. For example, the evidence is mixed on the relationship between issues and candidate preference (cf. [10] with [9] and [11]), as is the evidence on candidate qualities (cf. [13] with [14]). There is also evidence that voters take into account a candidate’s electability, or chances of winning the general election if he were to became the nominee [6], [13], [15]. However, the variable across all of the early literature with arguably the most consistent evidence is ideology. Specifically, Wattier [11] found that primary voters in the 1980 Republican primary tended to support the candidate who was closer to their own ideological identification, at least when they perceived an ideological difference between the candidates. Norrander [9] also found that ideology mattered to vote choice in the early 1980 Republican primaries (although not in the Democratic primaries). And somewhat more recently, Aldrich and Alvarez [14] found that ideology was a statistically significant predictor in many of the pairwise comparisons in both the Democratic and Republican primaries of 1988.

In addition, Aldrich and Alvarez [14] suggested a novel explanatory variable: policy *priorities*. They note that because primaries are intraparty contests, the political issues that usually divide the parties from each other “*should be* largely irrelevant in primary election decision making.” ([14], p. 290, emphasis in original). However, they argue that there is such a thing as policy priorities, meaning, the policy issues that each candidate chooses to emphasize or focus on. Aldrich and Alvarez present evidence that policy priorities influenced primary voting in the 1988 presidential primaries.

In sum, although there is evidence to suggest that ideology, candidate qualities, electability and issue priorities may each play some role in influencing individual primary voting, there is no one overarching explanation that consistently explains why an individual voter chooses one candidate over another in a presidential primary. In addition, none of the prior literature cited above has focused primarily on the role that religion plays in explaining individual vote choice among various candidates in presidential primaries. Only a few have included religion variables or related variables as independent variables at all. One of the exceptions to this is Aldrich and Alvarez [14], whose work is the most directly relevant here. First, one of the issue priorities they considered in the Republican primary was “moral values.” Their findings indicate that this was a statistically significant predictor of vote choice for each candidate (Bob Dole, Jack Kemp and Pat Robertson) compared to George H. W. Bush in the 1988 Republican primary. Specifically, voters for whom moral values were an important issue were more likely to vote for Robertson over Bush and for Kemp over Bush. The greatest effect was for Robertson. This would be as expected since Robertson was a well-known minister who discussed moral values issues more than any other candidate, and by a wide margin. Second, simply identifying as born again increased the probability (at the p < .001 level) of voting for each candidate compared to Bush, with the largest effect for Robertson.

Aldrich and Alvarez’s findings from two decades ago regarding “moral values” issues support the task here of looking more closely at the extent to which religion can help explain primary voting in more recent elections. In addition, work by Campbell, Green and Monson [16] concerning Mitt Romney’s Mormonism and the 2008 election provides fresh evidence that a candidate’s religious affiliation is most certainly relevant to voters’ evaluations of candidates. Although vote choice among various primary candidates was not the dependent variable being studied, Campbell et al. found—using experimental manipulations within surveys—that a voter’s degree of contact with and exposure to information about Mormons affected whether, all else equal, that voter would be more or less likely to vote for Romney [16]. The evidence presented below therefore extends and compliments the body of research cited above by focusing more specifically on the importance of religion variables in predicting vote choice at the individual level in recent Republican presidential primaries.

## Theory: *How* Might Religion Influence Presidential Primary Voting?

I argue that there are two primary ways in which a candidate in an American presidential election can make direct appeals to religious voters. First, the candidate can make explicit references to religion, faith or his own religious identity. For example, the candidate may explicitly discuss the importance of his religion or faith, and/or the candidate can explicitly and effectively label himself as “a Christian” or “born again.” In this way, the candidate is signaling to a specific religious constituency such as highly religious voters or born again Christians that “I am one of you” or “I understand you.” Put another way, as Popkin’s famous vignette of Gerald Ford illustrates, religious voters are watching to see if the candidate knows “how to eat a tamale” [12], but with the signal being religious in nature. Alternatively, or in addition to the first method, a candidate can explicitly discuss political issues that are intimately connected to certain religious beliefs, which attracts the attention of religious voters for whom those issues are often crucial. In the context of American politics over the last several decades, the two most prominent such “values issues” are abortion and same-sex marriage.

In sum, I theorize that a candidate’s use of these tactics are two of the primary ways in which candidates can appeal directly to religious voters, whether in the general election context or the primary election context. This is not to suggest that that these are the *only* two ways that religion can be relevant to one’s candidate preference. For example, other scholars have performed survey experiments to investigate the use of implicit, “coded” language that is designed to appeal to one constituency (such as evangelicals) without alienating another (moderates) [17]. My focus here, however, is on the use of *explicit* appeals to religious voters by candidates in American presidential primary elections, whether by explicit references to religion or by explicit references to political issues that are deeply connected to religion.

Given the mechanism explained above, variables such as a voter’s degree of religiosity or a voter’s own religious identification should have a positive effect on vote choice for the candidate who most explicitly appeals to religious voters. Put more specifically, I hypothesize that if a particular candidate explicitly makes appeals based on religion while other candidates do not, or if that candidate makes explicit religious appeals more frequently compared to other candidates, then one’s degree of religiosity or identification with a particular religious group should be a statistically significant predictor of voting for that particular candidate vis-à-vis each of the other candidates.

In order to test these hypotheses, the statistical analysis below is divided into two main categories. First, because of the extensive use and reliability of “attendance at religious services” as a measure of religious commitment and as a predictor of vote choice in American presidential general elections, that religious “behavior” variable will be discussed in detail for each primary election cycle. A separate part of the analysis will focus on religious identity or “belonging” by comparing respondents who identify as born again or evangelical Christians compared to respondents who are Christians, but *non*-born again or evangelical. The decision to treat these as distinct variables is consistent with the practice of scholars of religion and politics in the American context, who have long noted that there are three distinct (although not completely unrelated) aspects of religion that “have had important consequences in American political life: belonging (affiliation with a religious community), behaving (engaging in religious practices), and believing (holding religious beliefs). Each of these elements can play a role in determining how and to what extent a person’s religion influences his or her political views, affiliations, and activities.” [18], p. 13. In this data set, we have the ability to measure two of those three: “belonging” (born again/evangelical identification) and “behaving” (attendance at religious services), and because they are measuring two qualitatively different aspects of religion, they will be addressed in separate analyses. In addition, separating out the born again/evangelical identification variable will allow us to add to the prior research that evaluates whether those particular Republican identifiers were less likely to support Mitt Romney, the first viable Mormon candidate for president.

## The Effect of Religious Services Attendance in 2008

### Methods

In determining which Republican candidate in 2008 most explicitly (or most frequently) appealed to religious voters, I analyzed two data sources: (1) nationally-televised presidential debates; and (2) the candidates’ television ads. (See S1 Appendix for links to the publicly available sources for this data.) First, I read and analyzed the full text of the six nationally-televised Republican debates that took place immediately prior to Super Tuesday on February 5, 2008. This included the last debate before the Iowa caucuses, two debates in New Hampshire, one debate in South Carolina, one debate in Florida, and the final debate in California prior to Super Tuesday. Consistent with the theory outlined in the previous section, I looked for and counted any *overt* appeals by the candidates to religious voters, whether by explicit references to religion, faith, God, etc., or by emphasis on issues such as abortion and gay marriage. The list of words, phrases and references I considered using this criteria is as follows: God, Jesus, Christ, the Lord, Christian(s), Christianity, the Bible, scriptures from the Bible, evangelical(s), religion, faith, the Creator, “family values,” abortion, “right to life,” “sanctity of human life,” gay marriage, traditional marriage, and/or same-sex marriage. I limited my analysis to the final four candidates standing—Huckabee, McCain, Romney and Paul—as those were the candidates remaining in the race when the Pew Pre-Super Tuesday poll was taken. (Note: Paul was not present for one of the debates in New Hampshire; otherwise, all four candidates took part in these debates.) Admittedly, many of the words and phrases I chose are specific to the *Christian* tradition. However, all of the final four candidates in 2008 and 2012 identified as Christian, as did 87% and 84% of Republican respondents in the Pew Pre-Super Tuesday polls in 2008 and 2012, respectively.

I conclude from this full-text analysis that Huckabee most explicitly appealed to religious voters. First, neither McCain nor Paul made references to religion, faith, abortion or gay marriage in these debates. Second, Romney did talk about abortion or his faith in five of the six debates; however, in four of those five, he was on the defensive. This was not surprising, for a couple of reasons. First, Romney had been accused of being a “flip-flopper” and changing his position on abortion. And in fact, when he was running for governor of Massachusetts in 2002 (only six years before running for president), he stated in a televised debate that “I will preserve and protect a woman’s right to choose.” Given the fact that opposition to abortion is in the Republican party’s platform, this prior statement was problematic for Romney when he was seeking to win the Republican nomination for president. In addition, as Campbell, Green and Monson point out, many Christians are skeptical of Mormonism, and do not consider it a Christian religion [16]. The combination of these two things set up the dynamic of Romney being on the *defensive* when it came to religion (as opposed to his choosing affirmatively to bring up religion for his benefit), as the specific examples below demonstrate.

In the first debate (in response to a slight made against him by Alan Keyes), Romney stated that he became pro-life and as governor was pro-life. In the third debate, in response to a question suggesting that Romney was a “flip-flopper,” he admitted that he had changed his position on abortion. In the fourth debate, Romney had to explain why abortion was covered under the universal health care system in Massachusetts. In the fifth debate, Romney had to respond to a question on his identity as a Mormon, and in response to a question about his changing positions over the years, he reiterated that he is pro-life and stated that he is opposed to same-sex marriage. In the sixth debate, he took the opportunity—in response to a question of whether Ronald Reagan would vote for him—to state that he was pro-life and favored an amendment to protect marriage. In sum, in four of the five cases, Romney was not affirmatively appealing to religious voters; instead, he was *defending* himself, responding to comments that called into question his conservative credentials on abortion or that raised his Mormon faith as an obstacle to his winning the nomination.

In contrast, Huckabee was the only one of the four candidates who made an explicit reference to religion, faith, God, abortion or gay marriage in *each* of the six debates. In the first debate, in response to a question about one of his campaign ads in which he explicitly referenced his faith, Huckabee quoted the words of Christ from the Bible and the “endowed by our Creator” language from the Declaration of Independence. In the second debate, Huckabee noted that Americans’ rights don’t come from government, but from God. Also, in response to a question posed to each candidate—what distinguishes him from Barack Obama—Huckabee was the only candidate to mention the “sanctity of life” and same-sex marriage. In the third debate, in response to a question asked of all the candidates about why he was the best person to be the nominee, Huckabee was the only candidate who mentioned the “sanctity of human life.” In the fourth debate, Huckabee noted that Republicans should stick to their principles, including the sanctity of human life and the primacy of traditional marriage. He also mentioned being part of the evangelical coalition that helped elect Ronald Reagan. In the fifth debate, in response to a question highlighting criticism by some of Huckabee for using his faith, Huckabee said that faith has always been an important part of this country and that most Americans believe in God. He concluded by saying that if the voters want a president who doesn’t believe in God, they should “pick somebody else.” Finally, in the sixth debate, Huckabee was asked about whether Justice O’Connor was a good choice by President Reagan. In response, he emphasized the importance of the Republican position on abortion, noted that he was pro-life, and said that he would make every decision always on the side of life, without equivocation.

Given: (1) Huckabee’s *explicit* references to God, Christ, the Creator, and his faith, as well as the fact that he affirmatively took opportunities to bring up abortion and gay marriage; (2) Romney’s defensive posture when discussing religion and abortion; and (3) the lack of religious appeals from McCain or Paul, I conclude that Huckabee is the Republican candidate who most explicitly (and frequently) appealed to religious voters in the national debates immediately prior to Super Tuesday.

As a cross-check on my conclusion from the debates, I also analyzed a completely unrelated data set: the television ads aired by these four candidates prior to Super Tuesday. A review of that data reinforces the conclusion that Huckabee most explicitly appealed to religious voters. (The source for these ads is Stanford University’s Political Communication Lab database; see S1 Appendix for the link to this publicly available data.) Romney ran over forty television ads prior to Super Tuesday. Only *one* of his ads (representing 2% of his ads) focused on abortion or gay marriage. Aside from that ad, Romney did *mention* traditional marriage, being pro-life, or moral values in 15% of his ads, but even in those ads, other topics were the main focus. Romney also mentioned being pro-life and supporting traditional marriage in two other ads. However, those were attack ads on Huckabee, in which Romney attacked Huckabee on the issues of immigration and crime, and Romney’s reference to being pro-life and supporting traditional marriage were to make the point that he has the same views as Huckabee on those issues. In contrast, the dominant themes in 60% of Romney’s ads were either the economy/taxes, immigration and/or foreign policy.

Of Paul’s twelve television ads, only one was explicitly focused on abortion and the right to life, and one other mentioned that Paul was a “defender of life.” However, 75% of Paul’s ads were about taxes, military policy or immigration. Of McCain’s twenty-four television ads run prior to Super Tuesday, 25% focused on foreign policy or the military. Other ads ran the gamut, and included anti-Romney ads, ads about McCain’s leadership qualities, and ads vouching for McCain’s toughness and willingness to take unpopular stances. In only one ad did McCain make an oblique reference to “faith in God,” and in one other ad abortion was mentioned—but even that was as a means of attacking Romney. Thus, like Romney, neither Paul nor McCain was focused on religion.

However, of the eleven ads that Mike Huckabee ran prior to Super Tuesday, four focused on taxes and/or abolishing the IRS, but an equal number (36%) discussed his faith, God, Christ, abortion or gay marriage. In addition, two of those ads were the most explicitly *religious* ads run by any of the four candidates. For example, in one of his earliest ads, Huckabee says that his faith doesn’t just influence him, it defines him. He also states his belief that life begins at conception, and his graphics include the words “Christian leader.” Huckabee also ran an ad in December, reminding voters who were weary of the political campaigns that what really matters is the “celebration of the birth of Christ.” Neither McCain nor Romney nor Paul aired ads with the kinds of *explicit* references to religion and Christianity in Huckabee’s ads, or in which religion was the focus of the ad.

Thus, my analysis of the ads reinforces the conclusion that Huckabee was the candidate who most explicitly and consistently made religious appeals. In light of this evidence from the debates and ads, my hypothesis regarding religious services attendance is that as a voter’s level of attendance increases, so should his or her preference for Huckabee compared to each of the other candidates. I test this hypothesis on a Pew Research Center survey conducted from January 30, 2008, to February 2, 2008. This was just before Super Tuesday on February 5, 2008, and after the withdrawal of every major Republican candidate except for Huckabee, McCain, Romney and Paul. (In the case of Rudy Giuliani, part of the survey could have been contemporaneous with his withdrawal, which was announced on January 30, 2008. However, he was not included as one of the named answer choices for Republican primary respondents.) The sample had an original N of 1,502, with 455 Republican or Republican-leaning respondents expressing a preference for one of these four Republican candidates as their “first choice” for president. This survey was sponsored by The Pew Research Center for the People & the Press and was conducted by Princeton Survey Research Associates International. The data were made available through The Roper Center for Public Opinion Research, Cornell University, and the data set is entitled “Pew Research Center Poll # 2008-02ECO: February Economy Survey [USPEW2008-02ECO].” The Pew Research Center bears no responsibility for the analyses or interpretations of the data presented here. (Note: because the two surveys analyzed in this article were conducted for The Pew Research Center by Princeton Survey Research Associates International, the author presumes that these two reputable entities followed appropriate ethical guidelines and obtained appropriate consent for the surveys. See http://psrai.com/datacollection.shtml and http://psrai.com/about.shtml.)

While religious services attendance is my primary variable of interest, I include self-reported ideology in my model as a control variable because of the prior studies indicating that ideology is a statistically significant predictor of vote choice in presidential primaries. I also include the following standard independent variables that were available in this data set: income, education, gender, age, race, and region. (See S2 Appendix for how the independent variables were coded.)

### Results

**Fig 1:**
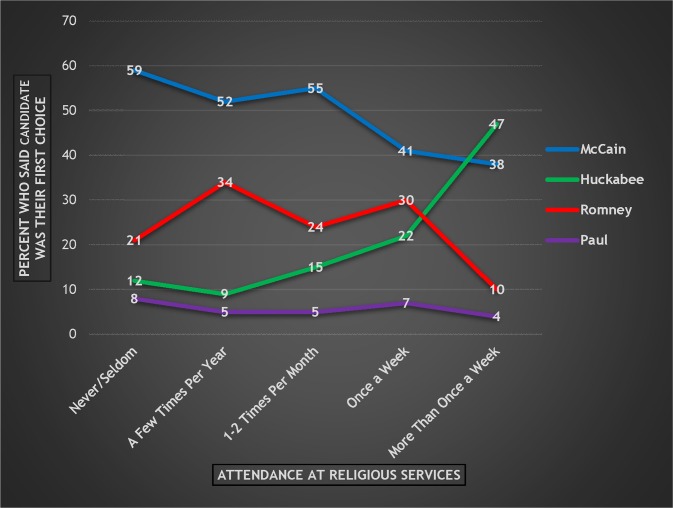
Respondents’ Preferred Candidate by Attendance at Religious Services, 2008 Republican Primary

In the Pew Pre-Super Tuesday survey, Republican and Republican-leaning respondents were asked which Republican candidate would be their first choice for president. Fig 1 presents each Republican candidate’s average level of support within each of the five different categories of religious services attendance. (The *overall* level of support for these four candidates among Republican or Republican-leaning respondents is as follows: McCain 42%; Romney 22%; Huckabee 20%; and Paul 6%. The percentages do not equal 100% because of respondents who were coded as “don’t know” or “refused.” Those respondents are not included in the rest of the analyses below.)

The first thing of note is that while McCain is the preferred candidate at four of the five levels of attendance (consistent with his status as the overall front-runner), his lead decreases substantially from the lowest level of attendance to the highest (from 59% to 38%). But perhaps the most striking thing is the trajectory of Huckabee’s support. Among Republican respondents who seldom or never attend religious services, Huckabee registers a low 12%. However, Huckabee’s support increases among moderate and high attenders, and ultimately reaches an impressive 47% among those who attend religious services more than once a week. Notably, Huckabee even surpasses McCain among the highest attending respondents. In addition, Huckabee’s percentage of support increases dramatically between the “Once a Week” and “More Than Once a Week” categories (from 22% to 47%). Therefore, Huckabee benefitted dramatically from increased religiosity among Republican respondents, and was the clear choice among those at the highest level of attendance. This is as predicted, as Huckabee was the candidate who most explicitly appealed to religious voters.

But how did attendance affect support for Romney—a Mormon candidate in a party heavily influenced by white evangelical Protestants, a strong plurality of whom (45%) reported in 2007 that they do not believe that Mormons are Christians [4]? Interestingly, Romney’s support is steady across the first four attendance categories, ranging between 21% and 34%. In fact, in each of those categories, Romney is the second choice behind McCain. However, in the highest attendance category, Romney’s support falls from 30% to 10%, well behind both McCain *and* Huckabee (and not much higher than Paul).

In order to more rigorously test these initial findings, I now turn to a multinomial logistic regression analysis of Republican primary candidate preference. As is clear from Table 1, when Huckabee is the base outcome, attendance at religious services is statistically significant in pairwise comparisons to *each* of the three other candidates. Specifically, as attendance at religious services increases, so does the likelihood of preferring Huckabee to McCain (p < .01), Huckabee to Romney (p < .001), and Huckabee to Paul (p = .053). However, when Romney is the base outcome, there is no difference based on attendance for Romney versus McCain or Paul. This suggests that highly religious respondents were not distinguishing Romney from McCain or Paul; however, they *were* making a distinction between Romney and Huckabee.

**Table 1:** 2008 Republican Primary Candidate Preference, Multinomial Logistic Regression (With “Attendance at Religious Services” Variable).

**Huckabee as the base outcome**			
	**McCain**	**Romney**	**Paul**
Attendance at Religious Services	-0.54** (0.19)	-0.65*** (0.19)	-0.46† (0.24)
Ideology	-0.92** (0.32)	-0.08 (0.31)	-0.18 (0.51)
Income	-0.09 (.11)	-0.06 (.11)	-0.17 (0.16)
Education	0.37† (.21)	0.40† (.22)	0.12 (.34)
Age	0.04** (.01)	0.04** (.01)	-0.02 (.02)
Nonwhite	1.88** (0.71)	1.23 (.79)	-13.32*** (.70)
Northeast	2.50** (.95)	2.18* (.99)	2.59* (1.1)
Midwest	0.34 (.43)	0.40 (.48)	-0.20 (.79)
West	0.68 (.54)	1.68** (.55)	1.30 (.94)
Sex	0.32 (.38)	0.39 (.39)	-0.39 (.58)
Constant	1.17 (1.54)	-1.09 (1.44)	2.65 (1.76)
**Romney as the base outcome**			
	**McCain**	**Paul**	
Attendance at Religious Services	0.11 (.12)	0.19 (.22)	
Ideology	-0.84*** (.22)	-0.11 (.49)	
Income	-0.03 (.07)	-0.11 (.15)	
Education	-0.03 (.15)	-0.28 (.32)	
Age	0.00 (.01)	-0.06*** (.02)	
Nonwhite	0.64 (.60)	-14.56*** (.74)	
Northeast	0.33 (.42)	0.41 (.77)	
Midwest	-0.06 (.41)	-0.60 (.82)	
West	-1.00* (.44)	-0.38 (.91)	
Sex	-0.07 (.31)	-0.78 (.57)	
Constant	2.26* (.98)	3.74* (1.73)	

Observations = 383; Pseudo R^2 = .16. Robust standard errors in parentheses.

***p< = 0.001

**p< = 0.01

*p< = 0.05

†*p* < .10 (p = .053)

**Fig 2:**
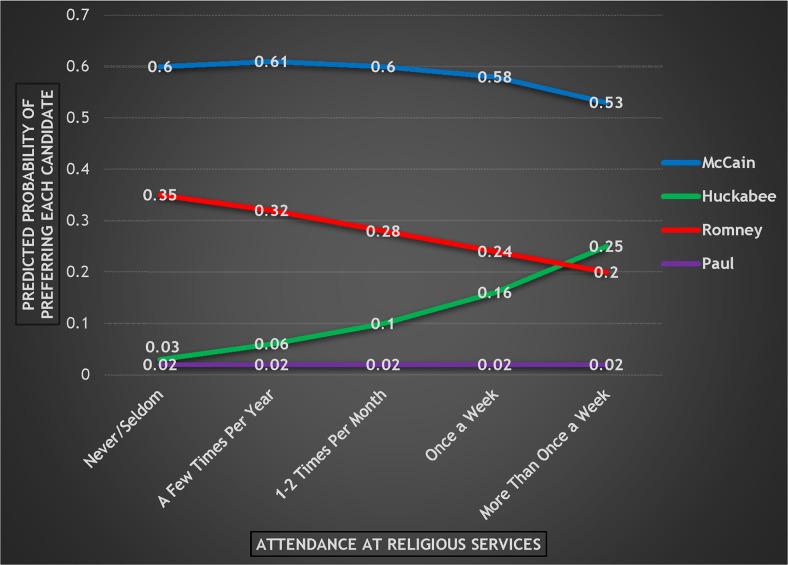
Probability of Preferring Each Candidate by Attendance at Religious Services, 2008 Republican Primary.

In order to turn these multinomial logistic regression coefficients into something more understandable, Fig 2 presents the predicted probabilities of preferring each candidate as attendance at religious services increases.

As attendance increases, there is a steady but small decline in the probability of supporting McCain (-.07), a steady and larger decline for Romney (-.15), and a significant increase for Huckabee (+.22). These results confirm that even with many other variables taken into account, increased attendance dramatically benefitted Huckabee—the candidate who most explicitly appealed to religious voters—and moderately harmed Romney—the candidate who was constantly on the defensive when it came to religion and “values issues.”

**Fig 3:**
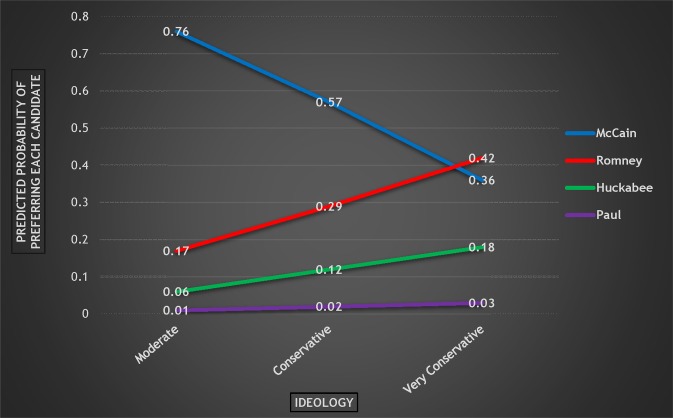
Probability of Preferring Each Candidate by Ideology, 2008 Republican Primary.

Other noteworthy results from these regressions include the importance of ideology, particularly when it comes to pairwise comparisons with McCain. As a respondent becomes more conservative, he is more likely to prefer both Huckabee to McCain and Romney to McCain. Increased conservatism therefore hurts McCain. There was no statistically significant difference based on ideology between Romney and Huckabee. As Fig 3 shows with predicted probabilities based on ideology, this leads to some interesting observations about the effect of ideology on candidate preference in 2008. The probability of preferring McCain plummets from .76 to .36 when going from “Moderate” respondents to “Very Conservative” respondents. And the biggest beneficiary of increasing ideological conservatism was Romney. As Republican primary voters became more conservative, the likelihood of preferring Romney increased dramatically (from .17 to .42), and among the most conservative voters, he was the preferred choice. This is important to keep in mind, as the results of increasing conservatism will be different for Romney in 2012.

In summary, attendance was a statistically significant predictor of candidate preference in pairwise comparisons between Huckabee and each of the other three candidates, and overall, increased attendance dramatically helped Huckabee but moderately hurt Romney. Therefore, attendance at religious services was important to understanding candidate preference in the 2008 Republican primary. In addition (and consistent with prior studies of primary vote choice), ideology was an important and statistically significant explanatory variable, with increased conservatism hurting McCain significantly, while benefitting Romney.

## The Effect of Religious Services Attendance in 2012

### Methods

To determine which candidate most explicitly appealed to religious voters in 2012, I again looked at two data sources: nationally-televised debates and the candidates’ television ads. First, I analyzed the full text from the eight Republican debates that took place prior to the start of the Pew Pre-Super Tuesday survey that I use as my data source for this analysis (starting with the debate in Iowa on December 11, 2011, and ending with the debate in Florida on January 26, 2012). As detailed earlier, I looked for and counted any appeals by the candidates to religious voters, whether by explicit references to religion, faith, God, etc., or by emphasizing issues such as abortion and gay marriage. Again, I limited my analysis to the final four candidates standing—Gingrich, Paul, Romney, and Santorum—as those were the candidates remaining when the Pew Pre-Super Tuesday poll was taken.

From my textual analysis I conclude that of these four candidates, Santorum is the one who most explicitly (and frequently) appealed to religious voters. This is based on the fact that Santorum was the candidate who either: (a) was the first to mention faith, family values, abortion, traditional marriage, etc.—*even though he was not prompted to by the question*; or (b) took an unrelated question and made it about one of these topics. This happened specifically in five of the eight debates analyzed. In addition, Santorum attacked Romney on more than one occasion for his record on abortion and gay marriage when Romney was the governor of Massachusetts. Santorum also attempted to distinguish himself from those who just “check [] boxes” or “whisper into the microphone” that they are pro-life, arguing that he has actually fought the battle to defend innocent human life. While Santorum did not make as many specific references to God or faith as Huckabee did in 2008, Santorum did explicitly make references to being a Christian and to Christ.

Although Romney did discuss issues such as abortion and gay marriage, it was often in response to a question in which his conservative credentials on those issues were being questioned or attacked. This happened in four of the eight debates. And while Gingrich made references in more than one debate to Christianity being under attack, Gingrich had to deal with questions about his marital history, which also put him on the defensive. Finally, Paul did not emphasize religion, and in one debate, when every candidate was asked pointedly how his religious beliefs would influence the decisions he made as president, Paul responded that his beliefs would *not* affect his oath of office as president.

An analysis of the ads that each candidate ran through February 7, 2012 (just before the Pew survey began), reinforces the conclusion that Santorum is the candidate who most explicitly made appeals to religious voters. (The source for these ads is Stanford University’s Political Communication Lab database; see S1 Appendix for the link to this publicly available data.) As of that date, Romney’s campaign had run thirty-eight television ads. *Not one* of them was expressly about “values issues” such as abortion and gay marriage. Romney had one television ad that made a general reference to “church” and “faith,” but the vast majority of Romney’s ads were about jobs, the economy and economic issues. In contrast, while Santorum’s campaign had run only six ads through February 7, 2012, four of these ads (67%) mentioned in some way “values issues,” Santorum’s faith, the sanctity of life, and/or abortion. Although three of Gingrich’s eighteen ads (16%) referenced faith or prayer or attacked Romney on abortion and gay marriage, in none of Gingrich’s ads was religion, abortion or gay marriage the focus. Finally, of Paul’s fifteen ads, two (13%) were very direct references to his being pro-life and against abortion in the context of his experience as an obstetrician delivering babies. However, the vast majority of Paul’s ads focused on the fact that he would reduce spending, balance the budget, cut taxes and bring the troops home so that American could stop policing the world.

In light of my conclusion that Santorum was the candidate who most explicitly appealed to religious voters, I now turn to results from 2012 Pew Pre-Super Tuesday poll, to see if increased attendance is associated with a preference for Santorum. The 2012 Pew poll was conducted from February 8 through February 12, 2012. This was before Super Tuesday on March 6, 2012, and after the withdrawal of every major Republican candidate except for Gingrich, Paul, Romney and Santorum. Prior to February 8, 2012, Romney had won three contests, Santorum had won four, and Gingrich had won one. Therefore, the primary was far from decided at that point. The sample had an original N of 1,500, with 491 Republican or Republican-leaning respondents expressing a preference for one of these four candidates as the candidate whom they would “most like to see nominated as the Republican party’s candidate.” This survey was sponsored by The Pew Research Center for the People & the Press and was conducted by Princeton Survey Research Associates International. The data were made available through The Roper Center for Public Opinion Research, Cornell University, and the data set is entitled “Pew Research Center Poll: February 2012 Political Survey [USPEW2012-02POL].” The Pew Research Center bears no responsibility for the analyses or interpretations of the data presented here. For consistency, the same control variables are included as in the 2008 regressions.

### Results

**Fig 4:**
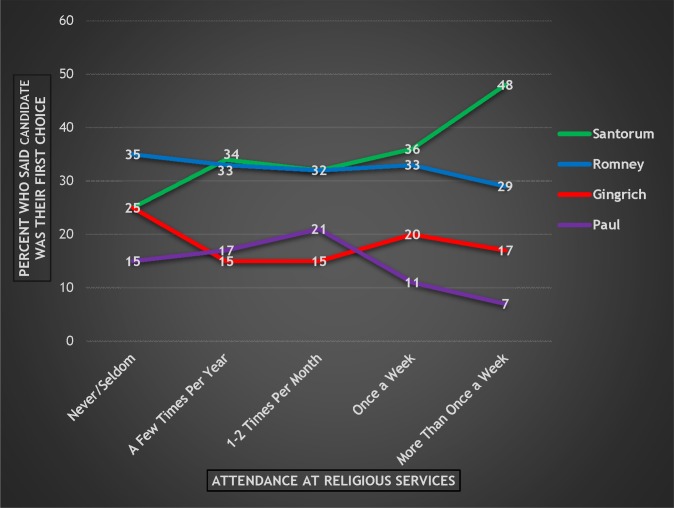
Respondents’ Preferred Candidate by Attendance at Religious Services, 2012 Republican Primary.

Fig 4 presents each candidate’s level of support across the five categories of attendance at religious services. (The *overall* level of support for these four candidates among Republican or Republican-leaning respondents is as follows: Santorum 30%; Romney 29%; Gingrich 17%; and Paul 12%. The percentages do not equal 100% because of respondents who were coded as “don’t know” or “refused.” Those respondents are not included in the rest of the analyses.)

Santorum has a clear upward trajectory as attendance increases, and is the preferred candidate at the two highest levels. Romney holds steady at the first three attendance categories, but loses out to Santorum by a slim margin among those who attend once a week. However, among those who attend religious services *more* than once a week, Santorum leads the others by a wide margin, including Romney by 20%. It is worth noting that this finding is consistent with evidence of *actual* voting in the 2012 Republican primaries taken from the state exit polls that contained measures of attendance; Santorum won the most frequent church attenders by a wide margin over Romney—57% to 26% [19].

Table 2 presents the multinomial logistic regression results.

**Table 2:** 2012 Republican Primary Candidate Preference, Multinomial Logistic Regression (With “Attendance at Religious Services” Variable)

**Santorum as the base outcome**			
	**Romney**	**Gingrich**	**Paul**
Attendance at Religious Services	-0.20† (0.10)	-0.18 (0.12)	-0.27* (0.14)
Ideology	-0.73** (0.23)	-0.48* (0.24)	-0.57* (0.27)
Income	-0.09 (.07)	-0.07 (.07)	-0.23* (0.10)
Education	0.42** (.14)	0.14 (.14)	0.17 (.22)
Age	0.00 (.01)	0.00 (.01)	-0.02* (.01)
Nonwhite	0.94 (0.55)	0.32 (.70)	1.89** (.66)
Northeast	-0.60 (.43)	-0.65 (.57)	-1.31* (.63)
Midwest	-0.35 (.37)	0.05 (.39)	0.23 (.43)
West	0.09 (.38)	0.35 (.45)	-1.88** (.67)
Sex	0.34 (.29)	0.29 (.33)	-0.10 (.37)
Constant	0.92 (1.14)	0.45 (1.03)	3.42** (1.25)
**Romney as the base outcome**			
	**Gingrich**	**Paul**	
Attendance at Religious Services	0.17 (.12)	-0.07 (.14)	
Ideology	0.25 (.26)	0.16 (.28)	
Income	0.02 (.08)	-0.14 (.10)	
Education	-0.29* (.14)	-0.25 (.22)	
Age	-0.00 (.01)	-0.03* (.01)	
Nonwhite	-0.62 (.64)	0.95 (.59)	
Northeast	-0.05 (.55)	-0.71 (.64)	
Midwest	0.40 (.42)	0.59 (.44)	
West	0.26 (.43)	-1.97** (.65)	
Sex	-0.05 (.34)	-0.44 (.37)	
Constant	-0.47 (1.08)	2.50* (1.24)	

Observations = 437; Pseudo R^2 = .08. Robust standard errors in parentheses.

**p< = 0.01

*p< = 0.05.

†*p* < .10 (p = .055)

**Fig 5:**
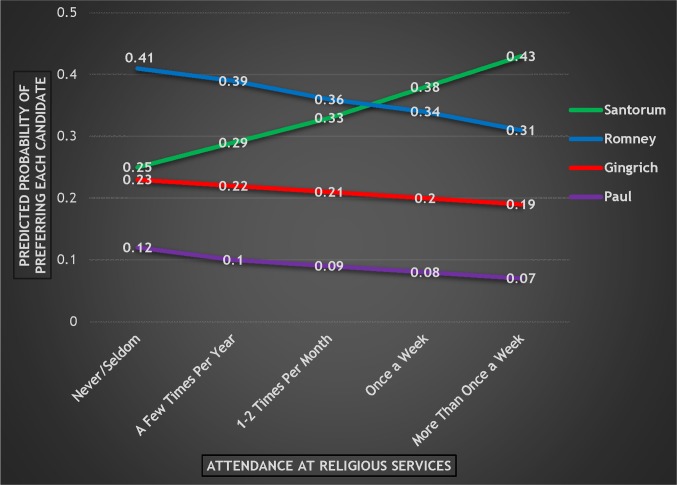
Probability of Preferring Each Candidate by Attendance at Religious Services, 2012 Republican Primary.

Attendance is statistically significant for Santorum versus Paul at the p < .05 level. Between Santorum and Romney, attendance is significant at the p < .10 level and is *just* outside of statistical significance at the .05 level (p = .055). This suggests that voters with higher degrees of religiosity were discriminating between Santorum and Romney and between Santorum and Paul, with increased levels of attendance benefitting Santorum. This suggestion is confirmed by converting the logistic regression results into predicted probabilities. As Fig 5 shows, there are moderate declines for Gingrich and Paul and a more substantial decline for Romney as attendance increases. On the other hand, the probability of preferring Santorum increases substantially from .25 to .43 when moving from the lowest level of attendance to the highest.

When comparing Romney’s performance in 2008 to Romney’s performance in 2012, the results are mixed. The good news for Romney is that the decline in his support as attendance increased was not as severe in 2012 (-.10) as it was in 2008 (-.15). In addition, Romney did much better in 2012 among those who attend at the *highest* level (the probability of this group preferring Romney in 2012 was .31 compared to .20 in 2008). This is evidence that Romney improved among this group, even with the presence of a strong religious candidate in Santorum. However, the bad news for Romney is that his support still declined overall as attendance increased and—perhaps more importantly—that Santorum surpassed Romney and became the preferred candidate among those attending once a week and among those attending more than once a week.

While Santorum had more support at every level compared to Huckabee in 2008, the trajectory of the line for Santorum in 2012 compared to Huckabee in 2008 is very similar. For example, Huckabee’s support increased by .22 while Santorum’s increased by .18 from the lowest level of attendance to the highest. This suggests that as a Republican respondent’s level of attendance increased, she was substantially more likely to prefer Huckabee in 2008 and Santorum in 2012—which is consistent with the hypothesis that voters with higher levels of attendance would prefer the candidate who most explicitly appealed to religious voters.

**Fig 6:**
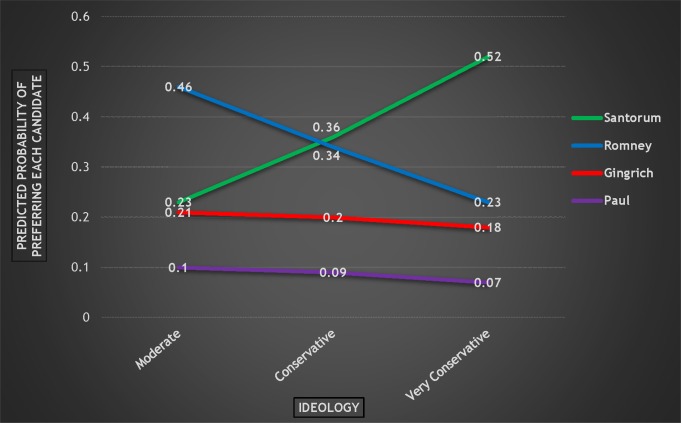
Probability of Preferring Each Candidate by Ideology, 2012 Republican Primary.

In addition, ideology was again a statistically significant predictor of candidate preference, at least when it came to Romney versus Santorum. As Fig 6 shows (somewhat dramatically), as respondents became more conservative, they were far more likely to prefer Santorum, and far less likely to prefer Romney. This is quite interesting since Romney was the preferred candidate in 2008 as conservatism increased. The most obvious explanation is that candidate preference is all relative when it comes to ideology; McCain perhaps was seen as the least conservative candidate in 2008, so *compared* to McCain, Romney was the choice of more conservative Republicans. However, with the very conservative Santorum in the race in 2012, Romney likely was not seen as conservative enough, and therefore, the most conservative Republicans did not prefer Romney.

In sum, while the results for attendance were not as dramatic in 2012 for Santorum as they were for Huckabee in 2008 (due in part to the fact that Santorum was a more popular candidate than Huckabee, both overall and at every level of attendance), there is confirmation of the hypothesis with regard to Paul versus Santorum, and weak confirmation (statistical significance at the p < .10 level and just outside the p < .05 level) that respondents were more likely to prefer Santorum to Romney as attendance increased. In addition, there is no doubt that the likelihood of preferring Santorum dramatically increased as attendance increased.

## The Effect of Self-Identification as a Born Again or Evangelical Christian

As a reminder, I argue that there are two primary ways that a candidate in an American presidential election can appeal to religious voters: the candidate can signal to a specific religious constituency such as highly religious voters or born again Christians that “I am one of you” or “I understand you” by making explicit references to religion or his own religious identity; and/or the candidate can discuss specific political issues that religious voters connect to their religious beliefs—the two most prominent of which in the American context are abortion and gay marriage. To the extent that any candidate explicitly sends these signals to a particular religious group, or does so more frequently than other candidates, then respondents who belong to that group should be more likely to vote for that particular candidate.

In light of this theory, my hypothesis for the 2008 Republican primary is that Huckabee will be the preferred candidate of Republican voters who identify as born again or evangelical, as not only was he a Baptist minister and therefore literally “one of them,” but also because (as discussed above) Huckabee was the candidate who most explicitly discussed his religion and the “values issues” that matter a great deal to born again voters. Although Romney was seen as personally very religious [4], his religious affiliation was potentially problematic. As Putnam and Campbell [20] explain, evangelicals are uneasy about Mormon religious beliefs, and “Mormonism is often labeled a ‘cult’ within evangelical circles.” [20], p. 502. In addition, Mormons are seen as a religious “out group” in the United States [16]. Therefore, it is highly unlikely that born again or evangelical Christians in 2008 viewed Romney as “one of them,” particularly given that 45% of white evangelical Protestants surveyed in 2007 did not even consider Mormons to be Christians [4]. And finally, as detailed above, Romney was constantly having to defend himself against charges that he was not conservative enough on issues such as abortion. McCain and Paul both identified as Christians; however, McCain was nominally religious and certainly did not focus on religion in his campaign, while Paul’s message was primarily focused on economic and foreign policy issues. Therefore, Huckabee stood out as the candidate with whom born again Christians would identify most as being “one of them.”

For 2012, the role of religious identification is a bit more complicated. Of the four candidates still in the race immediately prior to Super Tuesday, two were Catholic and one was Mormon. The only candidate who identified with a Protestant denomination was Paul; however, as detailed above, Paul’s message was focused on economic and foreign policy issues, *not* on religion or “values issues.” Gingrich was a converted Catholic, but he did not focus on religion, perhaps because he did not want to call attention to his own imperfect marital history. Regarding Romney—who again was on the defensive in 2012 when it came to “values issues”—there is no evidence that there was a marked change in evangelicals’ perception of the Mormon faith from 2008 to 2012. In fact, a Pew Research Center poll taken in 2011 indicates that a *majority* (53%) of white evangelicals who were Republicans or Republican-leaning did *not* believe that Mormons are Christians [21]. Thus, there was no reason to believe that born again or evangelical Republican voters in the 2012 election now saw Romney as “one of them.”

That left Rick Santorum. Despite Santorum’s Catholic identification, he *sounded* far more like a born again or evangelical Protestant. As described in detail above, Santorum consistently discussed his faith and the importance of protecting life and traditional marriage both in the debates and in his ads. In addition, Santorum’s family—who was often on stage with him at campaign rallies and during election results—was tangible evidence of the strength of his religious convictions. His wife, who has both a nursing degree and a law degree, left the workforce to raise and eventually home school their children. The Santorums have seven living children, including their youngest daughter, Bella, who was born with a severe genetic disorder. Bella was often present on stage in the 2012 campaign, and was also featured in a few of Santorum’s television ads. The message of Bella was clear: all life is precious, and must be protected.

Taking Santorum’s overt statements in the debates and his ads, as well as his family background into account, I conclude that Santorum was the candidate in 2012 who most clearly signaled to born again or evangelical Republican voters that he truly understands them and their concerns. Thus, despite the fact that his religious affiliation is Catholic, he “talked the talk” and “walked the walk” in a way that appealed to born again or evangelical Republicans, thereby signaling that he understood them and their values. As reinforcement for this conclusion, it is worth noting that when one hundred fifty evangelical leaders met in Texas prior to the South Carolina primary on January 21, 2012, in an attempt to coalesce behind one Republican candidate, Santorum eventually emerged as their choice [19].

In the 2008 and 2012 Pew Pre-Super Tuesday surveys, respondents were first asked what their religious affiliation was, if any. Those who responded “Protestant,” “Roman Catholic,” “Mormon,” “Orthodox” or who volunteered “Christian” (or who, after choosing “Something else” or “Don’t Know” responded “yes” when asked the follow up question of whether they thought of themselves as a Christian) were then asked “Would you describe yourself as a ‘born again’ or evangelical Christian, or not?” Based on these responses, of the 455 respondents in 2008 who expressed a preference for one of the four Republican candidates, 186 of them can be categorized as “born again Christians” and 214 can be categorized as “non-born again Christians.” (In the interests of using concise terminology, I refer to the “‘born again’ or evangelical Christian” respondents simply as “born again Christians.”) Of the 491 respondents in 2012 who expressed a preference for one of the four Republican candidates, 207 can be categorized as born again Christians and 207 can be categorized as non-born again Christians. Those are the two groups compared below (the few Republican respondents who identified as non-Christians are omitted).

**Fig 7:**
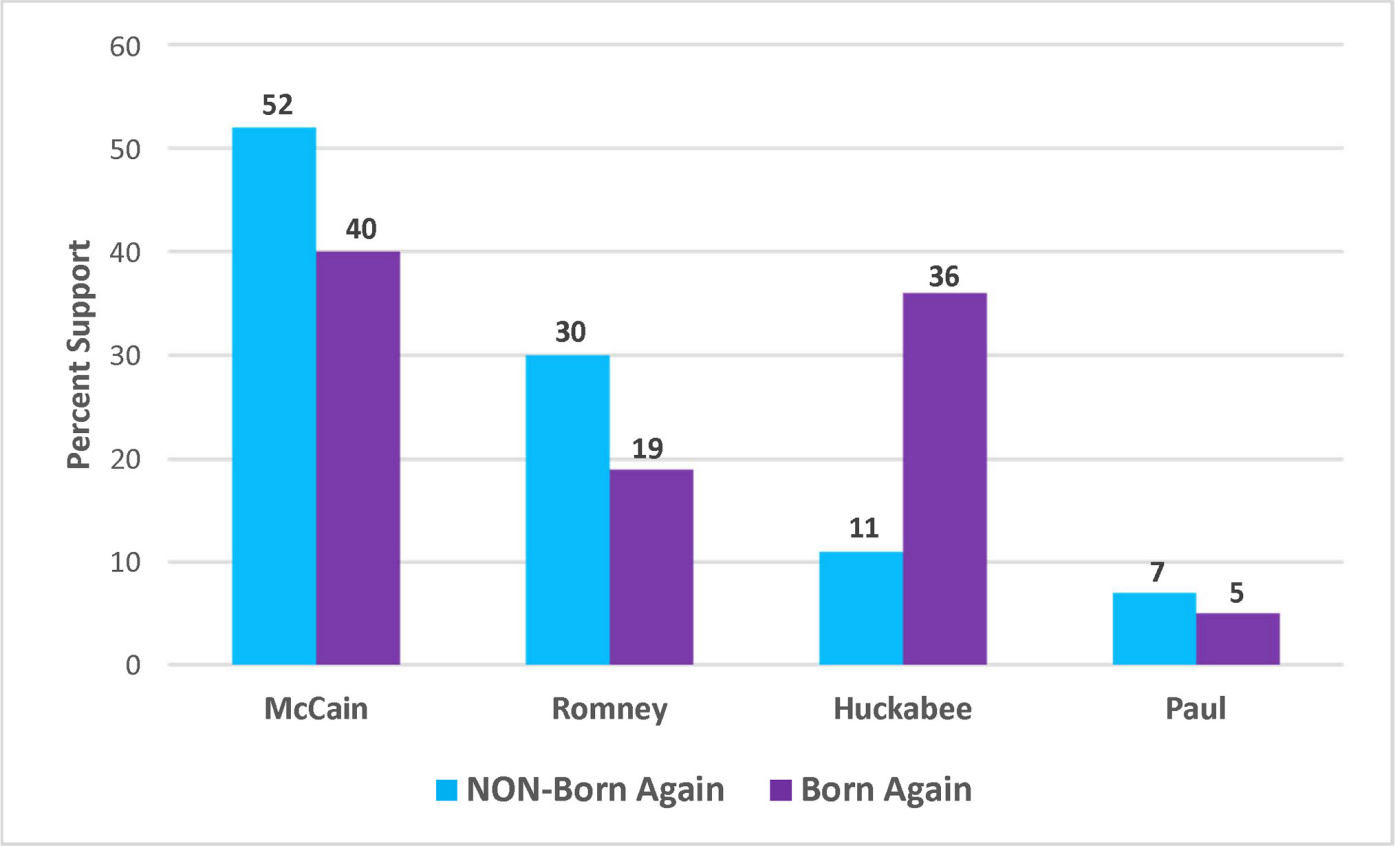
Difference in Levels of Candidate Support between Non-Born Again Christians and Born Again Christians, 2008 Republican Primary.

As is clear in Fig 7, in 2008, McCain was the clear favorite among Christian respondents who did *not* consider themselves born again or evangelical. Among born again Christians compared to non-born again Christians, McCain did 12% worse while Romney did 11% worse. Huckabee, on the other hand, did not do well at all among non-born again Christians. However, Huckabee’s support increased dramatically between non-born again Christians and born again Christians—from 11% to 36%. Although Huckabee still did not win a plurality among the born again Christian respondents, his 36% was a close second to McCain’s 40% among those who identified as born again; this is striking given that Huckabee trailed McCain among *all* Republican respondents by 42% to 22%.

Turning now to the multinomial logistic regression results in Table 3 below, when using the same independent variables as in Table 1 above, but using born again self-identification in the place of attendance, being born again was statistically significant between Huckabee and McCain at the p < .05 level (p = .013), between Huckabee and Romney at the p = .001 level, and between Huckabee and Paul at the p < .10 level (with born again identification making one more likely to prefer Huckabee to each of those candidates). Consistent with prior results, ideology was a statistically significant predictor in pairwise comparisons between Romney and McCain (p < .001) and between Huckabee and McCain (p < .01), with increasing conservatism once again hurting McCain.

**Table 3:** 2008 Republican Primary Candidate Preference, Multinomial Logistic Regression (With “Born Again Christian” Variable).

**Huckabee as the base outcome**			
	**McCain**	**Romney**	**Paul**
Born Again Christian	-1.06* (0.43)	-1.51*** (0.46)	-1.27† (0.68)
Ideology	-0.95** (0.33)	-0.13 (0.30)	-0.51 (0.53)
Income	-0.05 (.11)	-0.03 (.12)	-0.16 (0.18)
Education	0.23 (.23)	0.17 (.25)	0.04 (.38)
Age	0.04** (.01)	0.04** (.01)	-0.02 (.02)
Nonwhite	2.35* (1.09)	1.56 (1.10)	-14.09*** (1.09)
Northeast	4.07*** (1.08)	3.98*** (1.13)	3.81** (1.35)
Midwest	0.66 (.45)	0.68 (.53)	0.07 (.84)
West	0.50 (.57)	1.42** (.55)	1.49 (.97)
Sex	0.29 (.40)	0.24 (.42)	-0.53 (.64)
Constant	0.12 (1.57)	-1.97 (1.44)	2.53 (1.70)
**Romney as the base outcome**			
	**McCain**	**Paul**	
Born Again Christian	0.44 (.34)	0.24 (.66)	
Ideology	-0.82*** (.22)	-0.38 (.51)	
Income	-0.02 (.08)	-0.12 (.17)	
Education	0.05 (.17)	-0.13 (.35)	
Age	-0.00 (.01)	-0.06*** (.02)	
Nonwhite	0.79 (.58)	-15.64*** (.86)	
Northeast	0.09 (.46)	-0.18 (.96)	
Midwest	-0.01 (.44)	-0.61 (.88)	
West	-0.92† (.49)	0.07 (.96)	
Sex	0.05 (.32)	-0.76 (.63)	
Constant	2.08* (1.04)	4.50* (1.79)	

Observations = 339; Pseudo R^2 = .16. Robust standard errors in parentheses.

***p< = 0.001

**p< = 0.01

*p< = 0.05

†*p* < .10

When looking specifically at the predicted probabilities based on this regression in a pairwise comparison between Huckabee and Romney, being a born again Christian improved the probability of voting for Huckabee by 10%, and lowered the probability of voting for Romney by 12%.

**Fig 8:**
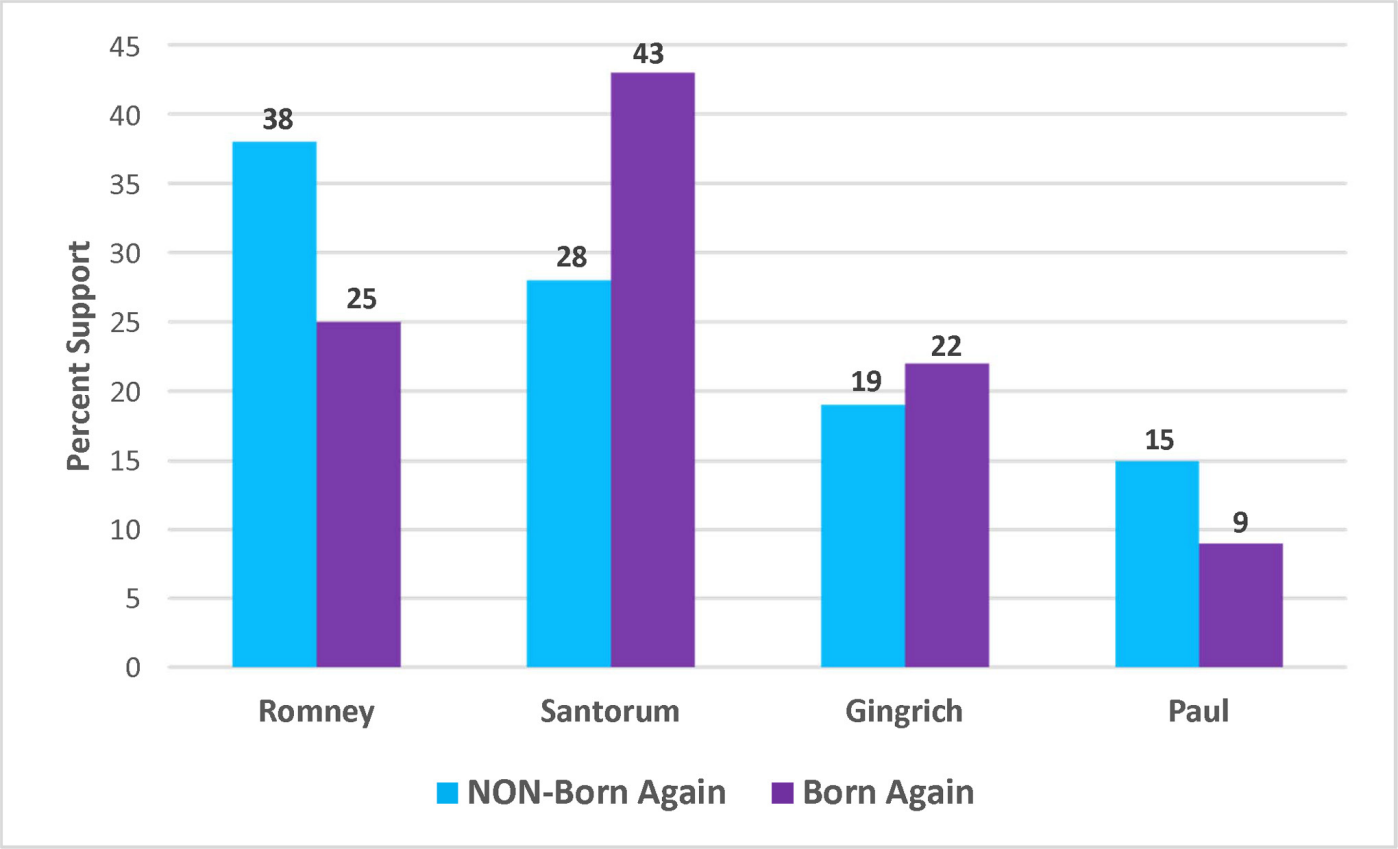
Difference in Levels of Candidate Support between Non-Born Again Christians and Born Again Christians, 2012 Republican Primary.

For 2012, in the crosstabs presented in Fig 8, there is support for the hypothesis that being a born again Christian would make one more likely to prefer Santorum. Specifically, between non-born again Christians and born again Christians, Santorum’s support increased by 15%. Romney’s support, however, decreased by 13%. Romney won a solid plurality of non-born again Christians (38%); however, Santorum’s numbers were not bad at 28%. When it came to born again Christians, Santorum won an impressive plurality with 43%, while Romney was a more distant second at 25%.

Multinomial logistic regression results are presented in Table 4 below. In pairwise comparisons in the multinomial logistic regression results, the only statistically significant result for the born again variable was between Santorum and Romney (p < .05). This confirms that born again Christian respondents were distinguishing between Santorum and Romney, in a way that benefitted Santorum. Notably, the one variable that was statistically significant (p < .05) in pairwise comparisons between Santorum and *each* of the other candidates was ideology, with increased conservatism benefitting Santorum.

**Table 4:** 2012 Republican Primary Candidate Preference, Multinomial Logistic Regression (With “Born Again Christian” Variable).

**Santorum as the base outcome**			
	**Romney**	**Gingrich**	**Paul**
Born Again Christian	-0.76* (0.32)	-0.17 (0.38)	-0.71 (0.44)
Ideology	-0.57* (0.25)	-0.54* (0.26)	-0.65* (0.31)
Income	-0.14† (.08)	-0.06 (.08)	-0.22† (0.12)
Education	0.29† (.16)	0.05 (.14)	-0.06 (.25)
Age	-0.00 (.01)	0.00 (.01)	-0.02 (.01)
Nonwhite	0.52 (0.60)	0.18 (.76)	2.47** (.80)
Northeast	-0.61 (.51)	-0.30 (.61)	-0.57 (.64)
Midwest	-0.44 (.39)	-0.08 (.42)	-0.12 (.50)
West	-0.00 (.40)	0.25 (.51)	-2.90*** (.85)
Sex	0.19 (.30)	0.14 (.35)	-0.25 (.41)
Constant	1.57 (1.12)	0.62 (1.04)	3.09* (1.33)
**Romney as the base outcome**			
	**Gingrich**	**Paul**	
Born Again Christian	0.60 (.40)	0.06 (.44)	
Ideology	0.03 (.29)	-0.07 (.32)	
Income	0.08 (.09)	-0.07 (.13)	
Education	-0.24 (.15)	-0.35 (.24)	
Age	0.00 (.01)	-0.01 (.01)	
Nonwhite	-0.34 (.69)	1.94** (.65)	
Northeast	0.31 (.61)	0.04 (.64)	
Midwest	0.36 (.46)	0.32 (.51)	
West	0.26 (.49)	-2.89*** (.80)	
Sex	-0.05 (.36)	-0.44 (.40)	
Constant	-0.95 (1.11)	1.52 (1.39)	

Observations = 368; Pseudo R^2 = .08. Robust standard errors in parentheses.

***p< = 0.001

**p< = 0.01

*p< = 0.05.

†*p* < .10

When looking at predicted probabilities based on the regressions, being born again results in a 12% increase in the probability of voting for Santorum, and a 14% decrease in the probability of voting for Romney when compared to non-born again Christians.

To summarize, in both 2008 and 2012, among born again Christians Romney lost out to the candidate who most explicitly discussed religion and/or values issues and thus communicated to born again or evangelical Christians that he was “one of them.” Specifically, there was a statistically significant difference in the probability of preferring Huckabee to Romney in 2008 and Santorum to Romney in 2012 for born again Christian respondents compared to non-born again Christian respondents.

## Discussion and Conclusion

In the 2008 Republican presidential primary, the candidate who most explicitly appealed to religious voters—Mike Huckabee—was the preferred candidate of Republican primary voters who attended religious services at the highest levels. In addition, when controlling for other variables, attendance at religious services was a statistically significant predictor of candidate preference for Huckabee versus *each* of the other three candidates; as attendance increased, so did the likelihood of preferring Huckabee to McCain, Huckabee to Romney, and Huckabee to Paul.

Similarly, in the 2012 Republican presidential primary, the candidate who most explicitly appealed to religious voters—Rick Santorum—was the preferred candidate of Republican primary voters who attended religious services at the highest levels. In addition, when controlling for other variables, attendance at religious services was a statistically significant predictor of candidate preference for Santorum versus Romney (p = .055) and for Santorum versus Paul (p < .05). While the results in 2012 were not as dramatic for Santorum as they were for Huckabee in 2008 (due in part to the fact that Santorum was a more popular candidate than Huckabee both overall, and at every level of attendance), the probability of preferring Santorum increased substantially as attendance increased, to the point that those attending at the highest levels had a probability of .43 of preferring Santorum—a probability that was higher than that for each of the other three candidates.

When it came to identifying as a born again Christian, those respondents preferred Huckabee to Romney, Huckabee to McCain, and Huckabee to Paul in 2008, and Santorum to Romney in 2012. Specifically with regard to Romney, being a born again Christian compared to a non-born again Christian resulted in a 12% decrease in the probability of preferring Romney in 2008, and a 14% decrease in 2012. Although these results of course are not definitive evidence of an anti-Mormon bias among born again Christians, they at least suggest that born again/evangelical Christians’ documented suspicions regarding Mormons [4], [16], [20], [21] may have come into play in both primaries.

Although religion, not ideology, was the focus of this paper, it is important to note that results in both 2008 and 2012 across different regressions confirm that ideology was a statistically significant predictor of candidate preference in many of the pairwise comparisons. These results therefore buttress prior research on the importance of ideology in explaining presidential primary voting.

Ultimately, the results presented in this article provide evidence that religion variables help explain candidate preference in the 2008 and 2012 Republican presidential primaries. This article therefore contributes to the literature on variables that are worth considering when attempting to explain presidential primary vote choice at the individual level, and also provides evidence that religion matters not only to voting in the general election, but in presidential primaries as well. These findings not only add to the extant literature on presidential primary voting, they also have implications for current, real-world politics. Candidates for the 2016 Republican presidential nomination clearly see religious voters in their party as important to winning the nomination, and were already courting this constituency as early as 2014 (before any of them officially announced), both at Ralph Reed’s Faith and Freedom Coalition conference and the high-profile Values Voter Summit, hosted by Tony Perkins and his influential, socially-conservative Family Research Council. Somewhat more dramatically, Senator Ted Cruz sent a clear signal to religious Christian voters by announcing his candidacy at Liberty University (the largest Christian university in the world, founded by Jerry Falwell), and then running his first campaign ad—heavy with religious references and imagery—on the weekend of Easter Sunday. Although at the time of this article’s publication it remains to be seen how the singular 2016 Republican primary will end, future research on this primary cycle will most certainly include an analysis of how highly religious Republican voters sorted themselves between Donald Trump—the iconoclastic and unconventional front-runner—and Cruz—one of the last candidates standing, and the candidate who arguably made the most explicit and consistent efforts to appeal to religious voters.

Finally, it is worth noting that Huckabee was the second-place candidate in 2008, and Santorum was the second-place candidate in 2012. Both gained momentum as the primary process moved along—particularly in the Deep South states, which contain large percentages of highly religious voters and born again Christians. Those facts, along with the findings of this paper, raise the question of whether a Republican candidate in future presidential primaries can effectively appeal to religious voters in the Republican party *early*—and more specifically, in the early primary states—so as to consolidate their support and gain traction against a more moderate Republican front-runner who is not as popular among highly religious Republicans.

## Supporting Information

S1 Appendix Links to publicly available sources for debate transcripts and television ads. (PDF)

S2 Appendix Coding of independent variables. (PDF)

S3 Appendix Confidence intervals for predicted probabilities, attendance at religious services, 2008 and 2012. (PDF)
