# Supplementary material for: The Effect of Religion on Candidate Preference in the 2008 and 2012 Republican Presidential Primaries
Source: PLoS One. 2016 Apr 4;11(4):e0152037. doi: 10.1371/journal.pone.0152037 (PMC4820110; doi:10.1371/journal.pone.0152037)
Supplement: S1 Appendix — (PDF) [file pone.0152037.s001.pdf]

S1 Appendix. Links to debate transcripts and television ads.

Debate Transcripts:

Many major news outlets prepare and publish written transcripts of primary debates, such as the networks that host the debates (e.g., ABC News, CNN, Fox News), as well as major American newspapers such as the New York Times and the Washington Post. The author did not rely on any *one* single news source for access to all of the transcripts analyzed.

One repository that provides transcripts of presidential primary debates all in one place is The American Presidency Project at the University of California, Santa Barbara. The link to that repository is: http://www.presidency.ucsb.edu/debates.php.

Television Ads:

The television ads were reviewed by going to the repository of ads for each election cycle compiled by Stanford University's Political Communication lab. The link to that repository for the 2008 election is: http://pcl.stanford.edu/campaigns/2008/primary/. The link to that repository for the 2012 election is: http://pcl.stanford.edu/campaigns/2012/primary/.
