# Supplementary material for: The Effect of Religion on Candidate Preference in the 2008 and 2012 Republican Presidential Primaries
Source: PLoS One. 2016 Apr 4;11(4):e0152037. doi: 10.1371/journal.pone.0152037 (PMC4820110; doi:10.1371/journal.pone.0152037)
Supplement: S2 Appendix — (PDF) [file pone.0152037.s002.pdf]

S2 Appendix

Coding of Independent Variables

Attendance at Religious Services, a 5-category scale ranging from "Never/Seldom" to "More than Once a Week."

Born again Christian, a dichotomous measure, separating those who are Christians *and* self-identify as born again or evangelical from those who are Christians but do not consider themselves born again or evangelical.

Ideology, a 3-category scale ranging from: "Moderate" to "Conservative" to "Very Conservative." (Note: Very Liberal and Liberal responses were collapsed with Moderates due to the extremely low numbers of Republican identifiers in those categories).

Income, a 9-point scale ranging from "Less than $10,000" to "$150,000 or more."

Education, a 4-point scale ranging from "High School or Less" to "Post-graduate Education."

Age, a continuous variable ranging from 18 to 93.

Nonwhite, a dichotomous variable, constructed from the race and ethnicity variable.

Sex, a dichotomous variable, coded 1 for female.

Region, divided into Northwest, Midwest, West and South, constructed from the census region variable.
