# Supplementary material for: The Effect of Religion on Candidate Preference in the 2008 and 2012 Republican Presidential Primaries
Source: PLoS One. 2016 Apr 4;11(4):e0152037. doi: 10.1371/journal.pone.0152037 (PMC4820110; doi:10.1371/journal.pone.0152037)
Supplement: S3 Appendix — (PDF) [file pone.0152037.s003.pdf]

S3 Appendix.

Confidence Intervals, Predicted Probabilities, Attendance at Religious Services 2008

	Predicted Probability	95% Confidence Interval
**Huckabee**		
Never/Seldom	.03	-.01, .07
A Few Times Per Year	.06	.01, .11
1-2 Times Per Month	.10	.04, .16
Once a Week	.16	.09, .23
More Than Once a Week	.25	.14, .36
**McCain**		
Never/Seldom	.60	.48, .72
A Few Times Per Year	.61	.52, .69
1-2 Times Per Month	.60	.54, .67
Once a Week	.58	.50, .66
More Than Once a Week	.53	.41, .65
**Romney**		
Never/Seldom	.35	.23, .47
A Few Times Per Year	.32	.24, .39
1-2 Times Per Month	.28	.22, .34
Once a Week	.24	.17, .31
More Than Once a Week	.20	.11, .28
**Paul**		
Never/Seldom	.02	.00, .04
A Few Times Per Year	.02	.01, .03
1-2 Times Per Month	.02	.01, .03
Once a Week	.02	.01, .04
More Than Once a Week	.02	.00, .04

Confidence Intervals, Predicted Probabilities, Attendance at Religious Services 2012

	Predicted Probability	95% Confidence Interval
**Santorum**		
Never/Seldom	.25	.16, .34
A Few Times Per Year	.29	.22, .36
1-2 Times Per Month	.33	.28, .39
Once a Week	.38	.32, .44
More Than Once a Week	.43	.34, .52
**Romney**		
Never/Seldom	.41	.30, .51
A Few Times Per Year	.39	.31, .46
1-2 Times Per Month	.36	.31, .42
Once a Week	.34	.27, .41
More Than Once a Week	.31	.22, .41
**Gingrich**		
Never/Seldom	.23	.14, .32
A Few Times Per Year	.22	.16, .28
1-2 Times Per Month	.21	.16, .25
Once a Week	.20	.14, .25
More Than Once a Week	.19	.11, .26
**Paul**		
Never/Seldom	.12	.05, .19
A Few Times Per Year	.10	.06, .15
1-2 Times Per Month	.09	.06, .13
Once a Week	.08	.04, .12
More Than Once a Week	.07	.03, .11
